# Combined genome-wide expression profiling and targeted RNA interference in primary mouse macrophages reveals perturbation of transcriptional networks associated with interferon signalling

**DOI:** 10.1186/1471-2164-10-372

**Published:** 2009-08-10

**Authors:** Paul Lacaze, Sobia Raza, Garwin Sing, David Page, Thorsten Forster, Petter Storm, Marie Craigon, Tarif Awad, Peter Ghazal, Tom C Freeman

**Affiliations:** 1 Division of Pathway Medicine, The University of Edinburgh, The Chancellor's Building, College of Medicine, 49 Little France Crescent, Edinburgh, UK; 2 Centre for Systems Biology at Edinburgh, The University of Edinburgh, Darwin Building, King's Buildings Campus, Mayfield Road, Edinburgh, UK; 3 Affymetrix Laboratory, Affymetrix, Inc. 3380 Central Expressway, Santa Clara, USA; 4 The Roslin Institute, University of Edinburgh, Midlothian, UK

## Abstract

**Background:**

Interferons (IFNs) are potent antiviral cytokines capable of reprogramming the macrophage phenotype through the induction of interferon-stimulated genes (ISGs). Here we have used targeted RNA interference to suppress the expression of a number of key genes associated with IFN signalling in murine macrophages prior to stimulation with interferon-gamma. Genome-wide changes in transcript abundance caused by siRNA activity were measured using exon-level microarrays in the presence or absence of IFNγ.

**Results:**

Transfection of murine bone-marrow derived macrophages (BMDMs) with a non-targeting (control) siRNA and 11 sequence-specific siRNAs was performed using a cationic lipid transfection reagent (Lipofectamine2000) prior to stimulation with IFNγ. Total RNA was harvested from cells and gene expression measured on Affymetrix GeneChip Mouse Exon 1.0 ST Arrays. Network-based analysis of these data revealed six siRNAs to cause a marked shift in the macrophage transcriptome in the presence or absence IFNγ. These six siRNAs targeted the Ifnb1, Irf3, Irf5, Stat1, Stat2 and Nfkb2 transcripts. The perturbation of the transcriptome by the six siRNAs was highly similar in each case and affected the expression of over 600 downstream transcripts. Regulated transcripts were clustered based on co-expression into five major groups corresponding to transcriptional networks associated with the type I and II IFN response, cell cycle regulation, and NF-KB signalling. In addition we have observed a significant non-specific immune stimulation of cells transfected with siRNA using Lipofectamine2000, suggesting use of this reagent in BMDMs, even at low concentrations, is enough to induce a type I IFN response.

**Conclusion:**

Our results provide evidence that the type I IFN response in murine BMDMs is dependent on Ifnb1, Irf3, Irf5, Stat1, Stat2 and Nfkb2, and that siRNAs targeted to these genes results in perturbation of key transcriptional networks associated with type I and type II IFN signalling and a suppression of macrophage M1 polarization.

## Background

Interferons (IFNs) are cytokines capable of causing a major shift in cellular gene expression through engagement of signal transduction pathways and subsequent activation of transcriptional networks. IFNs exert their multiple cellular effects through the induction of interferon-stimulated genes (ISGs) with antiviral, anti-proliferative and immunomodulatory properties. IFNs have proven useful clinically not only as potent agents against both RNA and DNA viruses, but also as response modifiers for oncology and as therapeutic agents for autoimmune diseases such as multiple sclerosis [1]. In spite of this our understanding of the signalling pathways and transcriptional networks associated with IFN signalling remains incomplete.

The production of type I IFNs (IFN-α, IFN-β, IFN-ω, IFN-ε or IFN-κ) is induced both *in vitro *and *in vivo *by the activation of the toll-like receptors (TLRs) [2-4] and other pathogen sensing systems (e.g. NOD receptors and RNA helicases RIG-I and MDA-5 [5]) through engagement with viruses, microbial products or other pro-inflammatory stimuli. Type I IFNs act through a common cell-surface receptor composed of two ubiquitously expressed transmembrane proteins, IFNAR1 and IFNAR2. IFNγ, the only type II IFN, shares little sequence homology with the type I IFNs and binds to a separate receptor complex [6]. Originally named 'macrophage activating factor', IFNγ is produced mainly by T-cells and natural killer cells. IFNγ is capable of inducing direct anti-microbial and anti-tumor mechanisms, up-regulating antigen presentation and arresting the cell cycle in macrophages and other cell types [7]. Macrophages activated by IFNγ are sometimes referred to as being polarized into an M1 phenotype [8,9].

Macrophages are primed for heightened immune activity by type I and type II IFNs through the transcriptional regulation of genes encoding receptors, transcription factors, cytokines, stress response proteins, immune signalling molecules and proteins with an anti-infective activity [10,11], thereby modulating the cell's antiviral and immuno-regulatory phenotype. Following ligand binding, engagement of common elements of the JAK-STAT signalling pathway i.e. Stat1, Stat2, Irf9 [12] and complexes thereof [13], leads to the activation of partially overlapping gene sets [14-16]. Crosstalk between the two IFN systems has been proposed as an evolved mechanism to help defend against a broader spectrum of pathogens [17], however the complexities of this signalling relationship remain poorly characterized. In this way, type I and II IFNs exert a pronounced and clinically important effect upon macrophages, a major effector cell of the innate immune system. Systems-level studies of macrophage activation to date have focused on network dynamics and *in silico *motif scanning to account the transcriptional complexity of the stimulated macrophage response [18-20]. These studies have proved useful in examining the dynamic nature of the macrophage transcriptome however they have not addressed specific roles of individual components within the IFN signalling system.

This study set out to investigate two aspects of the regulation of the macrophage phenotype by interferon. Firstly, a number of ISGs had been identified from previous experiments within our group as contributing to the protective effect of IFNγ during viral infection. These genes were identified from studies using siRNAs to target certain ISGs in mouse bone marrow derived macrophages (BMDMs) prior to infection with mouse cytomegalorvirus (mCMV). We observed significantly increased levels of replication of mCMV in BMDMs following treatment of the cells with a number siRNAs (data not shown) however the mechanisms by which the genes targeted contribute to the antiviral phenotype are largely unknown. Secondly, we have been interested in the IFN signalling pathways and the contribution of a number of cellular transcription factors to its regulation. We therefore wanted to investigate the contribution of these factors to the transcriptional response to IFNγ treatment.

In this study we describe the targeted knockdown of 11 genes using siRNA in BMDMs and the downstream changes in gene expression caused by specific siRNA activity as measured using genome-wide, exon-level microarrays (for list of genes targeted see Table 1). We chose to target a range of genes known to be involved in different aspects of the IFN response (both signalling and antiviral phenotype) based on the literature and our prior experimental observations [21]. Genes targeted included the type I IFN cytokine (Ifnb1), transcription factors with well-known roles in IFN signalling (Irf3, Stat1, Stat2), addition transcription factors with lesser known roles in IFN signalling (Nkfb2, Irf5), and a range of IFNγ-induced genes with known or putative antiviral function (Casp4, Ifi47, Lyn, Sod2, Traf1). These genes fall both up and downstream of IFNβ induction according to our knowledge of the IFN pathway [21] and include genes involved in both the type I and type II response.

**Table 1:** Panel of genes targeted by siRNA

**siRNA**	**Affymetrix Transcript ID**	**Refseq ID**	**Gene description**	**Relevance to IFNγ response**
**Casp4**	**6986649**	**NM_007609**	Caspase 4, apoptosis-related cysteine peptidase	pro-apoptotic, IFNγ induced
**Ifi47**	**6780707**	**NM_010999**	Interferon gamma inducible protein 47	IFNγ inducible protein
**Ifnb1**	**6923147**	**NM_010510**	Interferon beta 1, fibroblast	IFNγ induced, regulates immune signalling
**Irf3**	**6960326**	**NM_016849**	Interferon regulatory factor 3	virus-induced transcription factor, activates IFNα, & IFNβ
**Irf5**	**6945011**	**NM_012057**	Interferon regulatory factor 5	IFNγ induced, transcription factor, activates IFNα, IFNβ and plays a role in antiviral immunity & apoptosis
**Lyn**	**6911337**	**NM_001111096**	Yamaguchi sarcoma viral (v-yes-1) oncogene homolog	tyrosine kinase activity
**Nfkb2**	**6870063**	**NM_019408**	Nuclear factor of kappa light polypeptide gene enhancer in B-cells 2	DNA-binding sub-unit of NFkB transcription factor complex, regulates immune signalling
**Sod2**	**6858344**	**NM_013671**	Superoxide dismutase 2, mitochondrial	role in mitochondrial oxidative phosphorylation
**Stat1**	**6749376**	**NM_009283**	Signal transducer and activator of transcription 1	IFNγ-induced transcription factor, modulates IFN responses through signal transduction
**Stat2**	**6771641**	**NM_019963**	Signal transducer and activator of transcription 2	transcription factor, modulates IFN responses through signal transduction
**Traf1**	**6886021**	**NM_009421**	Tnf receptor-associated factor 1	mediates anti-apoptotic signals from TNF receptors

By suppressing the expression of these 11 genes we hypothesized that we would observe varying effects on the macrophage transcriptome, that when analyzed, would reveal functional insights into the activity of the encoded proteins. The study was also designed to provide a framework for beginning to test assumptions about literature based pathway-constructions [21], to assess the use of RNA interference as a tool for pathway analysis, and to test the performance of Affymetrix Exon Array 1.0ST platform. In addition, we have also examined in detail the non-specific inflammatory effect of siRNA transfection in murine BMDMs using a common lipid-based transfection reagent (Lipofectamine2000). Our findings highlight some of the limitations and technical issues associated with the use of RNAi technology in primary macrophages, and importantly also provide insights into factors contributing to the regulation of the transcriptional network associated with the type I and type II IFN response.

## Results

### Type I IFN response induced by non-targeting siRNA and lipid-carrier

**Figure 1:**
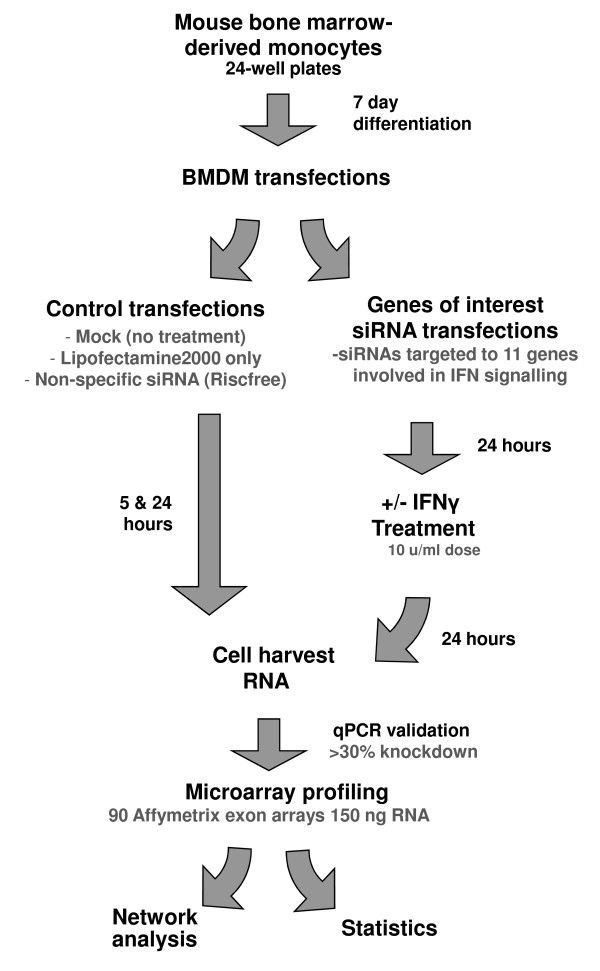
**Experimental Design**. Mouse bone marrow derived monocytes were cultured in the presence of CSF1 conditioned medium in six well plates for 7 days in order to allow differentiation into macrophages (BMDM). A series of control transfections were performed to assess the effect of Lipofectamine2000 and control RISC-Free siRNA. Six independent wells (on different plates) were then treated with either siRNAs targetting the mRNAs of one of 11 genes of interest or control siRNAs. 24 hours later three wells of each siRNA treament were stimuated by IFNγ and the cells were harvested 24 hours later. Total RNA was extracted and 150 ng labelled using whole transcript labelling and the samples run on Affymetrix mouse exon arrays. Data was then subjected to both network and statistical analyses.

siRNAs and the vectors used to deliver them have previously been shown to induce non-specific effects in cells and in particular to activate a type 1 IFN response [22-25]. In order to examine this affect in BMDMs we performed a series of mock transfections, treating cells for 24 hours with the cationic lipid reagent Lipofectamine2000 alone or combined with a non-targeting control siRNA (RISC-Free siRNA, Thermo Fisher). We used relatively low siRNA concentrations (20 nM) and low Lipofectamine2000 concentrations (0.2%) to replicate optimised experimental conditions used previously in our lab. All assays were performed in triplicate and total RNA was harvested at 5 and 24 hours post treatment and hybridized to Affymetrix GeneChip Mouse Exon 1.0 ST Arrays (Figure 1).

**Figure 2:**
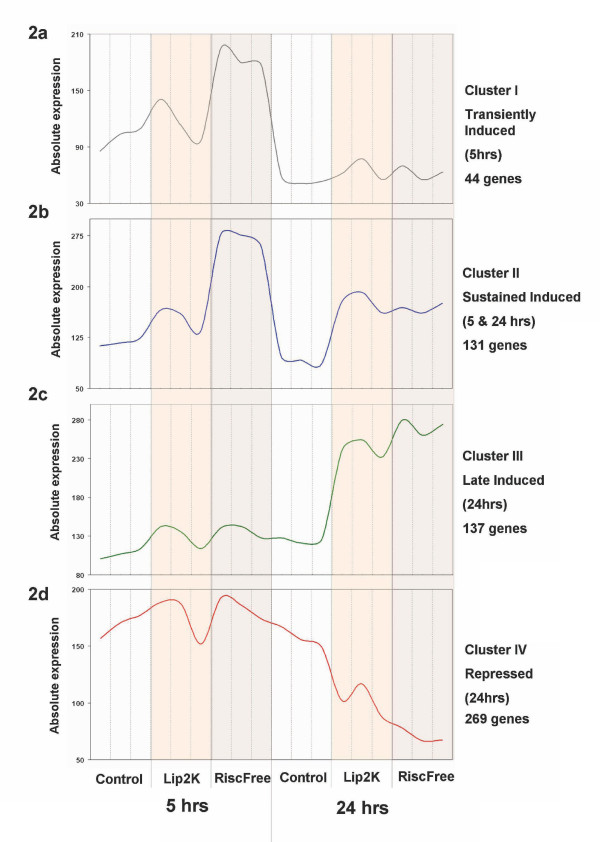
**Median profiles of co-expressed gene clusters**. Genes regulated by Lipofectamine2000 and RISC-Free siRNA mock transfections (triplicate arrays). For complete gene lists see Additional file 2.

We observed a marked up regulation of IFN-inducible transcripts at both 5 and 24 hours post-transfection of Lipofectamine2000/RISC-Free siRNA and by Lipofectamine2000 treatment alone. A total of 571 transcripts were differentially regulated by either of the two treatment conditions at either time point (ANOVA p < 0.01, fold change > 2). To visualize and further analyse the transcriptional response to Lipofectamine2000/RISC-Free siRNA and Lipofectamine2000 only treatments, the network analysis tool Biolayout *Express*^3D ^[26] was used to build graphs of the data. This software calculates the Pearson correlation between individual transcript profiles by drawing lines (edges) between genes (nodes) with related profiles and uses the MCL clustering algorithm [27] to divide the network into groups of genes with highly correlated expression profiles (for detailed description see Methods). The differently regulated transcripts could be divided into four clusters of co-expression (Figure 2a–e) reflecting different temporal and condition-specific profiles (see Additional file 1).

At the 5 hour time point we observed a transient up regulation of pro-inflammatory mediators including tumour necrosis factor alpha (Tnf) and chemokine Cxcl2 (Mip-2a) in response to transfection. This early response was stronger in the Lipofectamine2000/RISC-Free siRNA treatment group, but was also observed by Lipofectamine2000 treatment alone. Genes with an elevated expression at 5 and 24 hours were characteristic of a classic type I IFN response and included the interferon-inducible proteins (Ifi202, Ifi205, IIfi202b, Ifih1, Ifit1, Ifit2, Ifit3), members of the GBP protein family (Gbp1, Gbp2, Gbp3, Gbp5, Gbp6), antiviral genes (Mx2, Oas1g, Oas2, Oaslg), transcription factors (Stat1, Stat2) and chemokines (Cd40, Cxcl10, Ccl3, Ccl4). Another class of genes up-regulated at the 24 hour time point only (including C3, H2-A, H2-T22, Ifi203, Ifitm3, Ifi44, Irf7, Isg20, Oas3, Oasl2, Tlr9, and Zbp1) and also provided evidence of an a type I IFN transcriptional response. Over 250 genes were also down-regulated as part the response, including many cell-cycle regulators (for full gene lists see Additional file 2). The gene expression data indicated a substantial shift in the transcriptome of BMDMs caused by the lipid/RNA-mediated transfection process.

### Effect of IFNγ stimulation and sequence-specific siRNA transfection on the macrophage transcriptome

**Figure 3:**
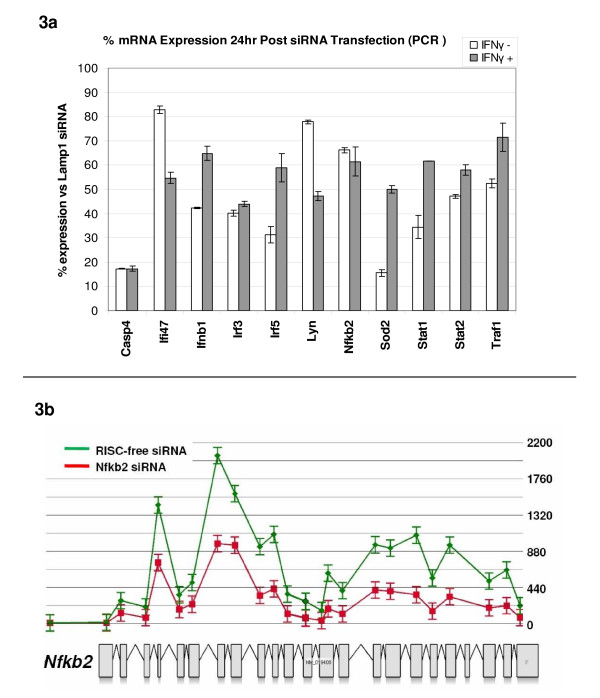
**a-b. qPCR and exon-level assessment of gene knockdown by siRNA**. 3a. Percentage mRNA knock-down 48 hours after siRNA transfection compared to a control siRNA tagetting the control gene Lamp1. 3b. Example of gene knock-down by exon array analysis. Level of knock-down at each of the 23 exon probesets across the entire length of the Nfkb2 transcript in the presence of IFNγ. The green line represents the median signal intensity in the three control arrays (RISC-Free) and red line the median signal intensity in the three Nfkb2 siRNA knock-down arrays (for other exon-level results see Additional file 3).

We next set out to investigate the effect of Lipofectamine2000 and targeting siRNA on murine BMDMs in the presence and absence of IFNγ stimulation. For these experiments, BMDMs were transfected in triplicate with one of a panel of 11 sequence-specific siRNAs and left for 24 hours (for a list of genes targeted see Table 1). IFNγ was added to half the samples for a subsequent 24 hours, after which the cultures were harvested (for experimental overview see Figure 1). RNA extracted from the samples was subjected to qPCR to evaluate the efficiency of gene knockdown in the presence and absence of IFNγ (Figure 3a). Where the target mRNA was knocked-down 30% or more on average in the presence of IFNγ the samples were taken forward for microarray analysis. In a number of cases genes of interest did not satisfy this criterion and no further analyses were performed. Total RNA from samples (in biological triplicates) that met the criteria were labelled and hybridized to Affymetrix GeneChip Mouse Exon 1.0 ST Arrays. The individual knockdown of target genes was also evaluated on microarrays at the transcript and exon level (Figure 3b and Additional file 3).

In order to assess the sequence-specific effects of siRNAs on the macrophage transcriptome we first performed a statistical analysis (for details see Methods). ANOVA analysis was used to detect statistically significant effects of siRNA and IFNγ as separate treatments across the entire data set. 242 transcripts were found to be differentially regulated between cells treated with RISC-Free siRNA alone and cells treated with RISC-Free siRNA and stimulated with IFNγ (p < 0.05, fold change > 2). Many genes up-regulated at 24 hours post-treatment with IFNγ are known interferon-stimualted genes and markers of a type II response [7]. These include the chemokines Cxcl9 (MIG) and Ccl2 (MCP-1), the co-activator of MHC class 2 genes (Ciita) and class II MHC components (H2-Aa/Ab1/DMb2/Ea/Eb2) and interferon regulatory factors (Irf1 and Irf8). Many other genes involved in antigen presentation were also up-regulated by IFNγ including components of the immunoproteasome (Psmb2/8/9/10) and Tap1/2 (for complete gene lists see Additional file 4). Some of the genes repressed by IFNγ treatment are known to be associated with cell cycle progression, checkpoint control, DNA synthesis and mitotic spinal formation, although many others are poorly annotated with little supporting literature. Overall, these data were consistent with our previous time-course microarray studies of IFNγ-stimulated BMDMs (available for download from ArrayExpress: E-MEXP-1490) [21], and with other previously published reports assessing the transcriptional response to IFNγ stimulation [15,16,28].

Transcripts regulated by sequence-specific siRNAs were detected by comparing expression levels between the non-targeting RISC-Free siRNA controls and each of the individual siRNA treatment groups. In the absence of IFNγ stimulation, 986 transcripts were found to be differentially expressed between the RISC-Free control and any of the 11 siRNA knock-down groups (p < 0.01, fold change > 2). In the presence of IFNγ stimulation, 892 transcripts were detected as differentially expressed following targeted siRNA treatment. 456 transcripts were found to be commonly affected by siRNA activity in both the absence and presence of IFNγ in at least one siRNA treatment (for statistically determined gene lists see Additional files 5, 6, 7).

**Figure 4:**
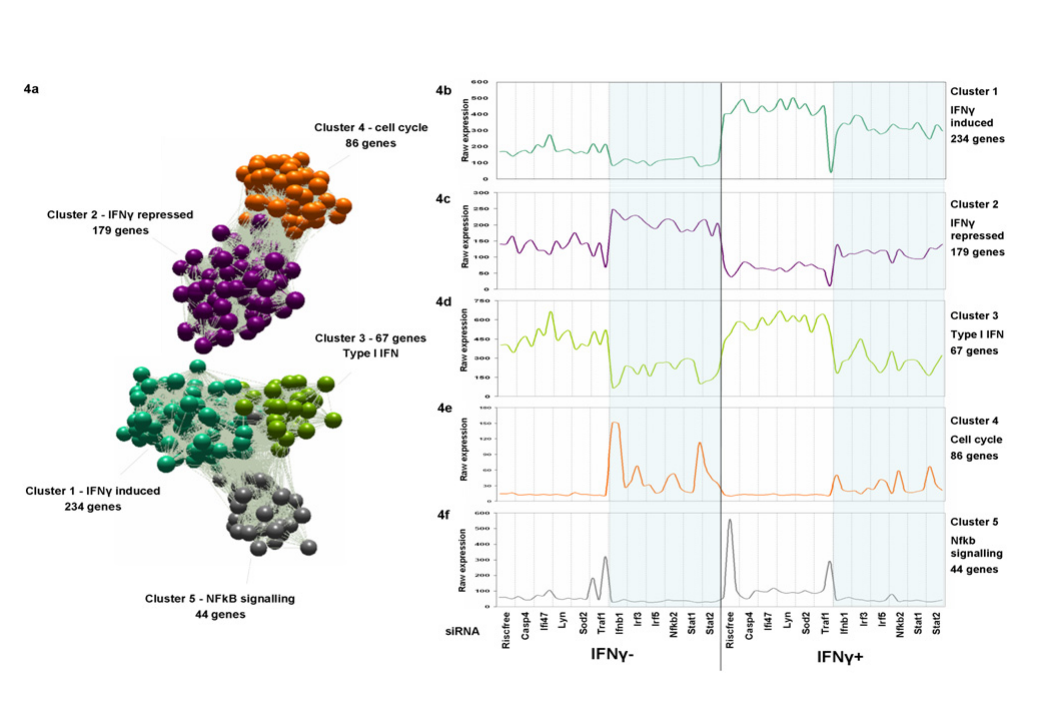
**a-f. Transcriptional network clustering of expression data from RNAi treated BMDMs – median expression profiles**. A network graph was clustered from microarray data using Pearson correlation r ≥ 0.9 & Markov clustering algorithm (MCL inflation value of 2.2). **4a: **Five main clusters of co-expression emerged containing genes influenced most by siRNA & IFNγ treatment. A consistent disruption of transcriptional activity of BMDM was observed using six particular siRNAs (shaded in blue) targeting Ifnb1, Irf3, Irf5, Nfkb2, Stat1 & Stat2 mRNAs. **4b**: Cluster 1 – 234 genes whose expression is induced by IFNγ and repressed by the six active siRNAs. **4c**: Cluster 2 – 179 genes repressed by IFNγ but de-repressed by six siRNAs. **4d**: Cluster 3 – 67 genes whose expression is not influenced by IFNγ at 24 hour assay point but repressed by six siRNAs. Many of these are innate immune response genes **4e**: Cluster 4 – 86 genes de-repressed by siRNAs, many of which have known functional association with cell cycle **4f: **Cluster 5 – 44 genes enriched with annotation for NF-kB signalling.

To visualize and further analyse the transcriptional response to siRNA and IFNγ treatments, the network analysis tool Biolayout *Express*^3D ^was again used to build graphs of the data. Five prominent clusters of co-expressed genes were identified from the RNAi data and formed a network graph containing all genes whose expression levels were altered most significantly by either IFNγ or siRNA treatments (see Figure 4a). We used a stringent Pearson cut off threshold of r = 0.9 and clustered the data using a MCL inflation value (which controls the granularity of clustering) of 2.2 to ensure minimal genes falling into these clusters by chance. The process resulted in a conservative total of 610 genes being included in the final network taken forward for further analysis. Each of the five major clusters of co-expressed genes that emerged from the network analysis had a distinctly different expression profile induced by the RNAi and/or the IFNγ treatment (Figure 4b–f).

Six siRNAs targeting the transcripts of the genes *Ifnb1, Irf3, Irf5, Stat1, Stat2 *and *Nfkb2 *were found to significantly perturb the transcriptional activity of primary macrophages. These siRNAs had a pronounced effect on the macrophage transcriptome in both the absence and presence of IFNγ stimulation, which was not seen in response to the five other siRNAs used in this study targeting *Casp4, Ifi47, Lyn, Sod2, Traf1 *transcripts. Each of the five gene clusters was mined for Gene Ontology (GO) and KEGG pathway enrichment to identify significant over representation of known biological and functional relationships shared by genes within a cluster using the DAVID online annotation tool [29]. Mean expression profiles of the clusters and the functional significance of the genes are summarized in Figure 4a–e and Table 2, respectively.

**Table 2:** Annotation for co-expressed gene clusters derived from RNAi and IFNγ treatments.

	**Cluster 1**	**Cluster 2**	**Cluster 3**	**Cluster 4**	**Cluster 5**
**Cluster designation**	IFNγ induced genes	IFNγ repressed genes	IFNβ induced genes	Cell cycle regulators	NFkB signalling genes

**No. genes in cluster (Pearson r = 0.9)**	234	179	67	86	44

**IFNγ treatment effect (at 24 hr assay point)**	Up-regulation	Repression	No effect	No significant effect	No significant effect

**siRNA effect (Ifnb1, Irf3/5, Stat1/2, Nfkb2)**	Repression	Up-regulation (De-represssion)	Repression	Up-regulation (De-reprsssion)	Repression

**Representative GO Terms (p < 0.05)**	Immune response, Antigen presentation	Intracellular signalling, Negative regulation of cellular process	Innate immune response, Inflammatory response	Cell cycle, DNA metablism, DNA replication	Stess response, Inflammatory response

**Enriched KEGG pathways (p < 0.05)**	TLR signalling, Cytokine-cytokine receptor, Cell adhesion molecules, JAK-STAT signalling	-	TLR signalling, Cytokine-cytokine receptor interaction	Cell cycle, DNA polymerase, Pyrimidine/purine metabolism	MAPK signalling, Apoptosis

**Promoter analysis (p < 0.05)**	ISRE sequence (34/234) NFkB targets (14/234)	-	ISRE sequence (16/67)	-	NFkB targets (12/44) CREB targets (4/44)

Representative gene membership*	Ccl2, Cxcl9, Cxcl11, Vegfa, Il15, Il18, Irf1, Irf2, Irf5, Ciita, Stat1, Stat3, Gbp1–5, Cd86, Itgb7, Cd274, Tlr6, Tlr9, Nod1, H2-Aa/H2-Ab1/Dmb2/Ea	Cd28, Cd33, Cd5l, Cdk6, Cebpa, Socs6, Igf2, Pfcd4, Dusp7	Isg20, Ifit1, Ifit2, Ifih1, Oasl1, Oas3, Mx1/2, Myd88, Tlr3, Ccl5, Ccl7, Ccl12, Stat2, Tnfrsf1a	Ccne1/2 Ccna2, Cdc2a, Cdca5, Cdca8, Cdc45l, Chek1, Brca1, Mcm2/3/4/5/6/7/10 Pola1, Pold1/e/e2	Nfkb1/2, Nfkbia/z, Ikb, Il1a/b, Tnf, Cxcl1/2, Nos2, Socs3, Tnfaip2/3

**Genes regulated by Lipofectamine2000 & non-specific siRNA transfection**	52/234 22.2%	10/179 5.6%	46/67 68.7%	69/86 80.2%	10/44 22.7%

*For full gene membership see Supplementarty table 2.

### Description of co-expressed gene clusters

*Cluster 1 *was the largest cluster in the data set consisting of 234 genes. The expression of these genes was up-regulated by IFNγ treatment (Figure 3b) and in most cases suppressed by the six siRNAs targeting Ifnb1, Irf3, Irf5, Stat1, Stat2, Nfkb2, both in presence and absence of IFNγ relative to the control and other siRNA treatments. Many of the genes within this cluster are consistent with an interferon signalling response [7] (see Table 2) and have immunomodulatory properties (Irf1, Stat1/3 Nod1, proteosome components Psmb8/9/10, Tap1/2). GO annotation mining showed them to be enriched in genes associated with toll-like receptor signalling (mmu04620), cytokine-cytokine receptor interaction (mmu04060), cell adhesion molecules (CAMs mmu04514), and JAK-STAT signalling (mmu04630) pathways. Interestingly, the genes in this cluster had a spectrum of expression from those whose expression was markedly up regulated by IFNγ treatment but little altered by the siRNAs e.g. Cxcl9 and genes associated with MHC class antigen presentation (Ciita, H2-Aa/Ab1/DMb2/EaEb2), ranging to those whose expression was only moderately up-regulated by IFNγ but significantly repressed by the six siRNAs (Ccl2, Gbp3/5, Il15, Il18, Tlr9). These genes are arranged at opposite ends of the cluster.

*Cluster 2 *contained a group of 179 genes with opposite profiles to *Cluster 1*. Their expression was repressed by IFNγ treatment but their basal level of expression was up-regulated when the cells had been treated with siRNAs targeting Ifnb1, Irf3, Irf5, Nfkb2, Stat1, Stat2 (Figure 3d). This cluster contained a number of negative regulators of cell growth and proliferation, and was enriched with GO annotations for biological processes including intracellular signalling (GO:0007242) and regulation of growth (GO:0045926). Overall however, the functional roles of many of the genes composing this cluster are poorly described.

*Cluster 3 *contained 67 genes with many known antiviral or antimicrobial effectors including the 2',5'-oligoadenylate synthetases (Oasl1, Oas3), TLR signalling components (Tlr3, Myd88), antiviral proteins Mx2, interferon-inducible genes Isg20, Isg54 (Ifit1) and Isg56 (Ifit2), p-56 related (Ifit) genes and chemokines (Ccl5, Ccl7, Ccl12). These genes reflected more of a type I IFN antiviral signature, with most transcripts involved in the innate immune response (for complete gene lists see Additional file 8). The expression of such type I IFN-induced transcripts should be low in inactivated cells, but we observed high levels of expression for these genes in all IFNγ un-treated and control RISC-Free samples. This suggested a pre-stimulation of cells occurring due to the transfection process, resulting in up-regulation of these transcripts. This was confirmed by the fact that many genes within this cluster were also up-regulated by Lipofectamine2000 and/or RISC-Free siRNA transfection in our mock transfection experiments (46/67 – 68.7%). Interestingly, these genes did not respond significantly to the subsequent IFNγ stimulus i.e. they were not differentially regulated at 24 hours post-IFNγ treatment. Furthermore, genes in this cluster were found to be strongly suppressed by the activity of Ifnb1, Irf3, Irf5, Stat1, Stat2 and Nfkb2 siRNAs (see figure 4d). In fact this group of genes were the most markedly effected of any genes in the data set by siRNA treatment (see Figure 4). This suggested that their expression was highly dependent on Ifnb1, Irf3, Irf5, Stat1, Stat2 and Nfkb2.

*Cluster 4 *contained 86 genes that were either not expressed or expressed at low levels in control samples, but were highly expressed (seemingly de-repressed) in samples treated with Ifnb1, Irf3, Irf5, Stat1, Stat2 and Nfkb2 siRNAs (especially in the absence of IFNγ treatment). The absolute level of expression of these genes varied greatly between technical replicates, yet the correlation of expression profiles within the cluster remained high. Annotation for this cluster was highly enriched with genes associated with cell cycle progression e.g. (Cdc45l, Cdc6, Cdca5, Cdca8), cyclins (Ccna2, Ccne1, Ccne2), kinesins (Kif11, Kif20a, Kif23) and DNA polymerase subunits (Pola1, Pold1, Pole) (for full list see Additional file 9). GO categories DNA metabolism (GO:0006259), DNA replication (GO:0006260), and cell cycle (GO:0007049) were significantly over-represented in this cluster.

*Cluster 5 *contained 44 genes whose expression was mildly elevated by IFNγ treatment and on average subtly down-regulated by the six siRNAs (i.e. similar to Cluster 1). However unlike genes in Cluster 1, these genes displayed a high degree of heterogeneity in expression levels between replicates, yet still remained highly correlated. Annotation for this cluster suggested a strong over-representation of NF-kB pathway-related genes including NF-kB transcription factor and signalling components (Nfkb1, Nfkb2, Nfkbia, Nfkbiz, Ikb) and NF-kB induced cytokines and chemokines (Tnf, Il1a, Il1b Cxcl1, and Cxcl2).

### Promoter analysis of the five RNAi-derived clusters

Motif scanning for the presence of ISRE (Interferon-Stimulated Response Elements), GAS (Gamma-Activated Sites) and NF-kB (Nuclear Factor-kappa B) consensus binding sequences was performed for all 2 kb upstream promoter regions for genes affected by siRNA activity (see Methods). Transcription factor target gene databases were also searched to identify target genes regulated by STAT, IRF, ISGF3 (Stat1/Stat2/Irf9) or NF-kB transcription factor complexes (see Methods). The transcriptional regulatory information was added as annotation classes and tested for over-representation within the clusters (see Table 2). This revealed statistically significant over-representation ISRE promoter sequences for genes within Cluster 1 and Cluster 3 (p = 0.019 and p = 0.0015, respectively) suggesting direct IFN regulation, and significant over-representation of NF-kB and CREB target genes in Cluster 5 (p = 4.93 × 10^-11 ^and p = 0.011, respectively) suggesting a JAK-STAT-independent mechanism of regulation. Clusters 2 and 4 showed no enrichment for these transcription factor binding sites using these methods.

### Overlap of genes affected by mock transfection and siRNA/IFNγtreatment

Of the 608 genes derived from the RNAi clusters, a considerable overlap (30.7%,187/608) were also found to be affected by Lipofectamine2000 treatment and/or transfection using non-targeting RISC-Free siRNA in the mock transfections (see Table 2). Overlap between the two datasets was most pronounced in Clusters 3 (68.7%, 46/67) and 4 (80.2%, 69/86). These clusters were associated with type I IFN activity and cell cycle regulation respectively.

## Discussion

In this study we set out to explore a number of questions. Firstly, our interest in pathways underpinning macrophage activation [21] motivated us to want to analyse in parallel the contribution of a number of known factors to the IFNγ response in these cells. Secondly, previous RNAi studies in our laboratory had identified a number of ISGs as contributing to the enhanced antiviral state of IFNγ-primed BMDMs to mCMV infection. We reasoned that we may further our understanding of their mechanism of action by analysing the affect of their knockdown at the transcriptional level. Finally, we wished to explore the potential of using a combination of the recently available exon level microarrays and improved RNAi targeting capabilities to gain insights into the interferon signalling pathways. In order to address these questions, 11 genes were targeted with siRNAs followed by IFNγ treatment and microarray analysis on the Affymetrix mouse exon 1.0 ST array platform. This study provides one of the few reports investigating the utility of combining RNA interference with global transcript profiling in macrophages. Our results highlight the potential of the approach, as well as some of the associated difficulties in performing this work.

In carrying out this investigation, we had to contend with a number of technical issues. The induction of an IFN response by double stranded RNA has been shown to be an issue in a number of different cell types [23-25,30,31]. This problem is potentially exacerbated in dendritic cells and macrophages due to their expression of TLRs, RNA helicases and other pattern recognition receptors involved in the sensing of pathogen-associated molecular patterns (PAMPs). The immuno-stimulatory properties of Lipofectamine2000 and other cationic lipid-based reagents have also been documented previously [32,33]. In order to minimise these known effects we therefore used final lipid and siRNA concentrations lower than recommended by the supplier (Thermo Fisher). However as we observed even these 'mild' transfection conditions still induced a significant type I IFN response in BMDMs. This response was characterized by the up-regulation of pro-inflammatory cytokines, transcription factors and other IFN-induced genes between 5–24 hours post-transfection of control siRNA and represented a significant shift in the transcriptional activity of these cells. The use of of 1,2-dioleoyl-3-trimethylammonium-propane (DOTAP) lipid formations to transfect siRNA in mouse cells has also been shown to induce the type I interferon response [33,34] and the immuno-stimulatory properties of lipid-based plasmid DNA transfections are well documented (reviewed in [35]). Furthermore, we have detected up-regulation of IFN-induced transcripts in response to Dharmafect 1 (Thermo Fisher) 48 hours post treatment of mouse fibroblasts (NIH-3T3) (unpublished data). These studies support the notion that IFN stimulation by siRNA and transfection reagents may be a widespread effect occurring in a number of different cell types [36].

A second technical issue of our study was the relatively low and variable knockdown efficiencies achieved when performing transfections in BMDMs, as measured by qPCR and array analysis. This was due in part to the low concentrations of reagents used, but also to the generally low efficiency of DNA/RNA delivery by transfection to primary cells such as macrophages. Primary macrophages are known to be considerably more difficult to transfect than cultured cell lines [37], making efficient gene knockdowns difficult to achieve in this study.

Despite these technical issues, we generated high quality microarray expression data from targeted transfection studies which was analysed using a combination of conventional statistical and network-based approaches [26]. At the exon level we were unable to observe any convincing evidence for alternative splicing events between the comparisons and therefore all further examination of the data was restricted to gene level analyses. Using network analysis it was possible to visualise relationships between differentially regulated transcripts and cluster them into distinct groups based on the similarity of their expression profiles across samples.

Network analysis of the data identified five major groups, or clusters, of co-expressed genes that were regulated by siRNA treatment. Genes within each cluster were found to be biologically related according to functional annotation and transcription factor binding site analysis, and co-regulated by IFNγ and/or siRNA treatment. The median expression profiles between clusters were markedly different, representing five distinct transcriptional networks. However, across all clusters, we observed a strong influence from the activity of six siRNAs targeted to the *Ifnb1, Irf3, Irf5, Stat1, Stat2 *and *Nfkb2 *genes. These siRNAs induced a global change in the macrophage transcriptome altering the expression of several hundred downstream genes. This effect was not observed in response to treatment of the cells with the RISC-free control siRNA or the other five siRNAs used in this study (targeting the Casp4, Ifi47, Lyn, Sod2 and Traf1 transcripts). The analyses presented here suggest that Ifnb1, Irf3, Irf5, Stat1, Stat2 and Nfkb2 all contribute to the control of genes regulated by both classes of interferon.

Clusters 1 and 2 in the data set (see Table 2 and Figure 4a–e) represent genes directly induced or repressed by IFNγ treatment respectively. Genes within these two clusters were also influenced by the activity of the Ifnb1, Irf3, Irf5, Stat1, Stat2 and Nfkb2 siRNAs (to a varying extent). Our analysis of transcripts regulated by IFNγ stimulation was consistent with our previous time-course experiments (ArrayExpress: E-MEXP-1490) and with previous profiling studies in this area [15,16,28,38]. Genes up-regulated by IFNγ stimulation (Cluster 1) reflected a broad range of immunomodulatory function, including an up-regulation of class II antigen presentation capabilities through the co-activator of MHC class 2 genes (Ciita), and histocompatibility class II antigens. Up-regulation of chemokines, complement components (C3, C4), caspases (Casp1, Casp7), interleukins (Il15, Il18) and interferon-induced proteins was also observed (for complete list see Additional file 8). In contrast, Cluster 2 contains many genes that are known to be suppressed by IFNγ stimulation and whose basal and IFNγ-stimulated expression level increased following treatment with the six siRNAs relative to the controls and other siRNA treatments.

Cluster 3 was made up of a group of genes that did not respond significantly to IFNγ stimulation, but were markedly down-regulated by the activity of the six siRNAs mentioned above. Importantly, Cluster 3 contained many well known type I IFN anti-microbial effector genes encoding interferon inducible proteins and chemokines suggesting regulation by IFNβ. Furthermore, statistically significant over-representation of ISRE promoter sequences in the 5' flanking regions of these genes, again suggests a dependency on type I IFN regulation and the IFN-induced transcription factor complex, ISGF3. Transcripts within Cluster 3 therefore appear to have a strong transcriptional dependency on type I IFNs and were the most markedly down-regulated by siRNA treatment in the dataset. In contrast, genes with a dependency on type II IFN i.e. genes involved in MHC class II antigen presentation [9,39], were not affected in this same manner by the six siRNAs. Genes with a co-dependency on both type I and type II IFN, we believe, are those in the data set being regulated by both IFNγ and the Ifnb1, Irf3, Irf5, Stat1, Stat2 and Nfkb2 siRNAs.

Cluster 4 in the data set is made up almost exclusively of genes whose function can be associated with cell cycle progression. Many transcripts within Cluster 4 (69/86) were found to be down-regulated in our mock transfection experiments during immune stimulation by Lipofectamine and RISC-Free siRNA. This suggests that transcripts within this cluster, most of which are associated with cell cycle control, are down regulated or suppressed during macrophage activation. This was supported by very low expression levels for these transcripts observed in RISC-Free control samples 24 hours post IFNγ treatment in our second series of experiments. The expression of genes within this cluster was markedly induced (or de-repressed) in response to Ifnb1, Irf3, Irf5, Stat1, Stat2 and Nfkb2 siRNAs (see Figure 4e). This suggests a link between the six genes targeted and the control of the cell cycle, which we believe may be a secondary effect of disrupting the IFN pathway. Cluster 5 in the data set consists of a group of 44 genes, many associated with the NF-kB signalling system supporting a link between this system and the IFN pathway in BMDMs [40-42]. Details of this involvement are however ill-defined.

In trying to explain these observations regarding the Ifnb1, Irf3, Irf5, Stat1, Stat2 and Nfkb2 knockdown phenotypes, our hypothesis is that suppression of Ifnb1, Irf3, Irf5, Stat1, Stat2 and Nfkb2 using siRNA all result in a perturbation of the type I IFN response in BMDMs. We believe this occurs either by a direct perturbation of IFNβ induction following activation of pathogen detecting systems (as seen with Irf3, Irf5, Ifnb1 and Nfkb2 siRNAs) or by perturbation of signalling downstream of the type I receptor complex (as seen with Stat1 and Stat2 siRNAs). Perturbation at either of these levels in the pathway, we believe, is what accounts for the common downstream alteration of several hundred interferon-regulated transcripts as observed in this study. We also believe the perturbation has also influenced NF-kB signalling and resulted in a modulation of the cell cycle. The common phenotype induced by Ifnb1, Irf3, Irf5, Stat1, Stat2 and Nfkb2 siRNAs observed in our study suggests each of the genes targeted are operating at a similar level or hierarchy within the interferon pathway, and that suppression of these genes has a similar effect on the macrophage transcriptome.

**Figure 5:**
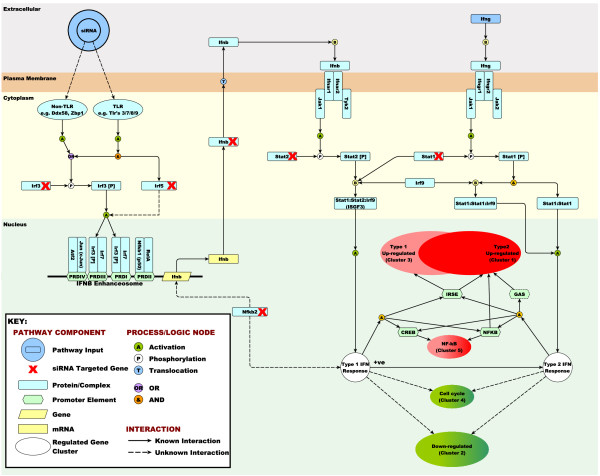
**Model of known components of the IFN signalling pathway and explanation of observed results**. Transfection of siRNA using Lipofectamine2000 in mouse BMDMs inductes a type I IFN response. This probably occurrs through the activation of pattern recognition receptors (PRRs) by dsRNA and/or liposome complexes. Downstream of PRR activation interferon regulatory factor 3 (Irf3) is phospohorylated and translocates to the nucleus where it which binds to the IFNβ promotor to induce expression of the IFNβ transcript (Ifnb1). It has been proposed that interferon regulatory factor 5 (Irf5) contributes in a similar manner to induce type I IFN during the antiviral response, and components of the NF-kB pathway are also known to contribute at this level. Once induced, the IFNβ acts in an autocrine manner to activate JAK-STAT signalling and subsequent formation of the interferon-stimulated gene factor 3 (ISGF3), a transcription factor complex composed of Stat1, Stat2 and Irf9 proteins. ISGF3 is responsible for driving the expression of type I interferon-stimulated genes via the ISRE (interferon stimulate response element) found within the promoter sequence of many ISGs affected in this study. Our data suggest that siRNAs targeted to the Ifnb1, Irf3, Irf5, Stat1, Stat2 and Nfkb2 transcripts all disrupt the type I IFN response at a similar level, and therefore alter the downstream expression of several hundred IFN-inducible genes in a similar way. Our data also suggest that type I IFN signalling strongly down-regulates cell cycle genes and influences the activity of the NF-κB signalling and many of the genes associated with a type II IFNγ response. Subsequent activation by IFNγ influences the expression of a different but significantly overlapping set of genes and the magnitude of this response is clearly influenced by the prior activation of the cells by type I signalling.

We have been modelling the IFN system based on findings reported in the literature [21] and have used this model to help further interpret the findings of this study (a simplified version of the model is shown in Figure 5). As the model indicates, macrophages possess many cell surface and intracellular receptors for the detection of a broad range of molecular species specifically found in pathogenic organisms. It is some of these receptors that are undoubtedly activated by the transfection reagents/siRNA. The most likely candidates are those with RNA binding function such as Ddx58 (RIG-I) and Ifih1 (Mda5) which detect cytoplasmic viral ssRNA and dsRNA [5,43] and/or the endosomal TLR receptors namely Tlr3, Tlr7 and Tlr8, that are also activated by these molecules [44]. It is possible that these receptors may be sensitive to synthetic siRNA in activating the IFN response. Other TLR receptors e.g. Tlr1/2 and Tlr2/6 that are known to sensitive to lipopeptides and peptidoglycans might additionally be activated by the transfection reagent. According to our model, activation of all of these receptors ultimately leads to the phosphorylation, dimerization and translocation of Irf3 and/or Irf7 to the nucleus where they activate Ifnb1 expression. This formation of Irf3:Irf3 and Irf3:Irf7 dimers is an important regulatory event during the induction of IFNβ [45] and subsequent up-regulation of ISGs following pattern recognition receptor activation [46,47]. Therefore the suppression Irf3 using siRNA would be expected to have a strong influence on IFN regulation and subsequent downstream ISG expression. Indeed this is what we observed. In a similar fashion, if the IFNβ (Ifnb1) transcript itself was targeted for suppression, it might also be predicted to have a direct effect on downstream expression of type I IFN-induced genes (as also observed in this study). From this perspective, the phenotypes observed in response to Irf3 and Ifnb1 siRNAs in this study are as expected.

Stat1 and Stat2 are primary transcriptional regulators of the IFN response and are essential components of the JAK-STAT signalling pathway. Their phosphorylation by ligand-activated interferon receptors leads them to form the Stat1-Stat2-Irf9 (ISGF3 complex) which is crucial for the transcriptional regulation of the IFN response via ISRE elements [48] following induction of IFNβ. Stat1 also homodimerizes following activation of the type II IFN receptor complex to form the Stat1:Stat1 AAF complex to induce further transcription via GAS sites [12]. The suppression of either Stat1 or Stat2 function would be predicted to affect IFN signalling directly and alter ISG expression by preventing transcription factor binding to ISG promoter sites. Indeed, we have observed a phenotype consistent with this in our study following Stat1 and Stat2 suppression using siRNA.

The role of the transcription factor Irf5 in the type I IFN response is less well established, although recently Paun *et al*. has demonstrated that murine Irf5 can be activated by both TBK1 and MyD88 to form homodimers which bind to and activate transcription of type I IFN and inflammatory cytokine genes [49]. Perturbation of Irf5 through siRNA knockdown in this study suggests that Irf5 could influence type I IFN-induced transcriptional networks at a similar level to Irf3. Further studies however will be required to clarify the role Irf5 in this context. Likewise, the role of Nfkb2 (p52/p100 subunit) in type I IFN signalling is difficult to explain based on the current understanding of this protein in the regulation of innate immunity. Nfkb2 is known to form transcription factor complexes with RelB and/or Bcl3 as part of the 'alternative' NF-KB pathway, often associated with B-cell maturation and lymphoid development [50]. Our study strongly suggests that Nfkb2 may play a central role in the regulation of the type I IFN response in mouse BMDMs, however this observation is only partially supported in the literature [41,51]. The presence of NF-kB binding elements in the IFNβ promoter (enhanceosome) [45] raises the possibility of a direct interaction of this protein in IFNβ regulation, however further studies will of course be necessary to support this hypothesis.

## Conclusion

Taken as a whole these data support the idea that many transcripts are regulated by both the type I and II IFN networks and co-stimulation has an additive effect in regulating their expression. In terms of the involvement of specific factors in regulating this pathway, then four of the genes studied here (Ifnb1, Irf3, Stat1 and Stat2) can be explained based on findings from previous studies and fit our model of events [21]. However, two genes, Irf5 and Nfkb2, do not fit this model and our findings indicate that they may play important but as yet uncharacterised roles within this pathway. What perhaps is surprising is that the absolute expression level of all of these factors would appear to influence the level of type I IFN signalling, indicating a level of co-dependency which we would not necessarily predicted. Furthermore, their marked influence on type I signalling raises the possibility that these genes might be targeted in order to suppress this pathway and ameliorate the non-specific immune-activation caused by siRNA delivery *in vitro *or *in vivo*.

## Methods

### Cell propagation and differentiation

Primary mouse monocytes were harvested from 10–12 week old male balb/c mouse bone marrow, re-suspended in DMEM-F12/10% FCS/10% L929 medium and plated in a 24-well plate at a concentration of 5 × 10^5 ^cells/well. To differentiate the cells from monocytes into primary macrophages, cells were then cultured for a further 7 days in DMEM-F12 growth media supplemented with 10% L929 conditioned medium which contains the macrophage stimulating factor CSF-1, with media changes on days 3 and 5. Flow cytometry was performed on day 6 confirming a double positive cell surface phenotype (> 99%) for F4/80 and CD11b macrophage markers (see Additional File 9).

### Transfection of siRNA and IFNγ treatment

siRNAs (SMARTpools, Thermo Fisher Inc, MA, USA) were purchased at a 5 nmol scale and redissolved in 1× siRNA buffer (Thermo Fisher Inc, MA, USA) to a final concentration of 1 μM. These contained 4 different siRNAs per pool each designed to target the same transcript. To transfect at a final concentration of 20 nM, 1 μl of siRNA SMARTpool was used with 49 μl of Optimem (Invitrogen, CA, USA) solution while 2 μl of Lipofectamine 2000 (L2K, Invitrogen, CA, USA) was mixed with 48 μl Optimem. Following incubation for 5 min, the siRNA mix was added to the L2K mix and incubated for a further 30 min, after which 400 μl of DMEM-F12/10% FCS/L929 medium lacking antibiotics was added to the siRNA:L2K complexes. Growth medium was removed and cells were washed in 1× PBS before 500 μl of the siRNA:L2K liposomes were added. Cells were then incubated for a further 24 h (37°C, 5% CO2). For IFNγ treatments, growth medium was replaced with medium containing 10 U/ml recombinant mouse IFNγ. Cells were cultured for a further 24 h prior to harvesting of total RNA.

### RNA extraction & quantitative real time PCR

Total RNA was extracted using an RNeasy Plus kit (Qiagen, Hilden, Germany) according to manufacturer's instructions. RNA was quantified and quality controlled using a NanoDrop spectrophotometer (NanoDrop Technologies, DE, USA) and BioAnalyser 2100 (Agilent, CA, USA). RT-PCR was performed on RNA samples diluted to 10 ng/μl using TaqMan primer/probe sets (Applied Biosystems, CA, USA) and Brilliant One-Step q-RT-PCR kit (Stratagene, CA, USA) according to manufacturer's instructions. Samples were analysed using a MXPRO3000P and MXPro software (Stratagene, CA, USA), respectively. Lamp1 was used as an internal control.

### Microarray target labelling and data processing

150 ng of total RNA was processed using the Exon array target labelling kit (Affymetrix, CA, USA) according to manufacturer's instructions for small sample labelling but without the use of the RiboMinus step. Quality control of microarray data was performed using Affymetrix Expression Console™ following standard Affymetrix Exon Array protocols. Data normalisation, statisical and exon-level analyses were perforemd using Partek Genomics Suite™ (MO, USA). All probesets from the 'core' set of exons were imported and normalized using gcRMA. An exon-level analysis was performed and each siRNA targeted transcript was assessed for silencing at the exon-level (supplemetary data). The data was also explored for evidence of alternative splicing events. Transcript level summarization was then performed using the mean of all probesets across each transcript. An expression level filter was applied to exclude all transcripts with a maximum raw signal intensity of less than 50 over across all arrays (11,286 transcripts). All microarray data used in this study is available for download from Gene Expression Omnibus (GSE14534) Furthermore, the data used for construction of the networks and the graphs themselves can be downloaded (Additional files 10, 11 from journal website).

### Statistical Analysis

All experimental conditions employed three biological replicates and these were used for statistical comparisons between groups. For mock transfections ANOVA testing was performed on all filtered probesets comparing untreated control samples vs. Lipofectamine2000 and vs. Lipofectamine2000:RISC-Free siRNA at both the 5 and 24 hour time points separately employing a < 0.01 p-value and > 2 fold change cutoff. For the second set of experiments target specific siRNA effects were detected by ANOVA between RISC-Free control samples and individual siRNA knockdown groups (< 0.01 p-value, > 2 fold change). IFNγ treatment effects were detected by ANOVA testing between untreated and IFNγ treated RISC-Free control groups.

### Network Analysis of Microarray Data

Tabulated normalised expression data with unique probe identifiers and annotation (nodeclass) columns was loaded into the application BioLayout Express3D [26]. An all-versus-all Pearson correlation matrix was then calculated based on the expression profile of all filtered probe sets. Pearson correlations greater than r = 0.7 were stored and network graphs were constructed at using different thresholds above this value. Graphs consist of nodes representing transcripts connected by edges respresenting expression correlations above the set threshold. The MCL algorithm clusters the network graphs according to the connectivity between nodes as defined by a mathematical bootstrapping procedure [27]. Network graphs were explored for clusters of genes whose expression profile was influenced by siRNA treatment and IFNγ treatment. The final layout and analysis of the data was performed using a Pearson correlation cut off of r = 0.9 and the resultant graph clustered using an MCL inflation value of 2.2. To gain an estimate of the statistical significance of a terms (nodeclasses) represented within a cluster, BioLayout Express3D uses a two-sided Fisher's exact test in a similar way to other methods (e.g., GoMiner [51]). A Bonferroni correction is also used to correct Fisher's p-values for multiple testing. This approach was used to examine the representation of specific regulatory sites assoicated with the gene clusters.

### Transcription factor binding site analysis

2000 bp upstream promoter sequences for all RefSeq 2007 genes were retreived from NCBI and BLAST searched for the following consensus transcription factor binding sequences using BioPerl; ISRE – GGAAANNGAAACT [52], GAS – TTCNNNGAA [53]. Target gene databases queried for Rel/NF-KB, STAT and IRF bound target genes; 'NFKB.org' – Gilmore Lab, Boston University , 'The Transcriptional Regulatory Element Database', Zhang Lab, Cold Spring Harbor  and 'Rel/NF-kappaB target genes' Gosselin, Touzet, Abbadie, Institut de Biologie de Lille et LIFL .

## Authors' contributions

PL contributed to experimental procedures, performed data analysis and was a primary author of the manuscript; SR, GS, DP, PS and MC all made significant contributions microarray processing and other laboratory procedures; TF carried out statistical analysis of the data; TA was instrumental in helping design the experiment and co-ordinating the collaboration with Affymetrix; PG led the discussions with the experiment's design and helped in the writing of the manuscript and TCF contributed with experimental design, oversaw experimental procedures and data analysis, and was a primary author of the manuscript.

## Supplementary Material

Additional file 1 **Mock transfection gene expression clusters.** Click here for file

Additional file 2 **Genes differentially regulated in mock transfections (571 genes).** Click here for file

Additional file 3 **Exon-level assessment of gene knockdown by siRNA**. Level of knock-down at each of the exon probesets across the entire length of transcripts in the presence of IFNγ. Green profiles represents the median signal intensity in the three control arrays (RISC-Free) and red line the median signal intensity in the three siRNA targeted arrays. Click here for file

Additional file 4 **Genes differentially regulated by IFNγ (242 genes).** Click here for file

Additional file 5 **Genes differentially regulated by targeting siRNAs in absence of IFNγ (986 genes).** Click here for file

Additional file 6 **Genes differentially regulated by targeting siRNAs in presence of IFNγ (842 genes).** Click here for file

Additional file 7 **Genes differentially regulated by targeting siRNAs in both the absence and presence of IFNγ (456 genes).** Click here for file

Additional file 8 **RNAi gene expression clusters.** Click here for file

Additional File 9 **Macrophage flow cytometry**. Mouse bone-marrow derived monocytes were stained on day 6 of differentiation for CD11b and F4/80 cell surface markers to identify the presence of macrophages. Over 99% of cells within the cultures are CD11b and F4/80 positive. Click here for file

Additional file 10 **BioLayout3D expression data from mock transfection study.** Click here for file

Additional file 11 **BioLayout3D expression data from targeted RNAi study.** Click here for file
