# Supplementary figures and images for: Combined genome-wide expression profiling and targeted RNA interference in primary mouse macrophages reveals perturbation of transcriptional networks associated with interferon signalling
Source: BMC Genomics. 2009 Aug 10;10:372. doi: 10.1186/1471-2164-10-372 (PMC2741489; doi:10.1186/1471-2164-10-372)

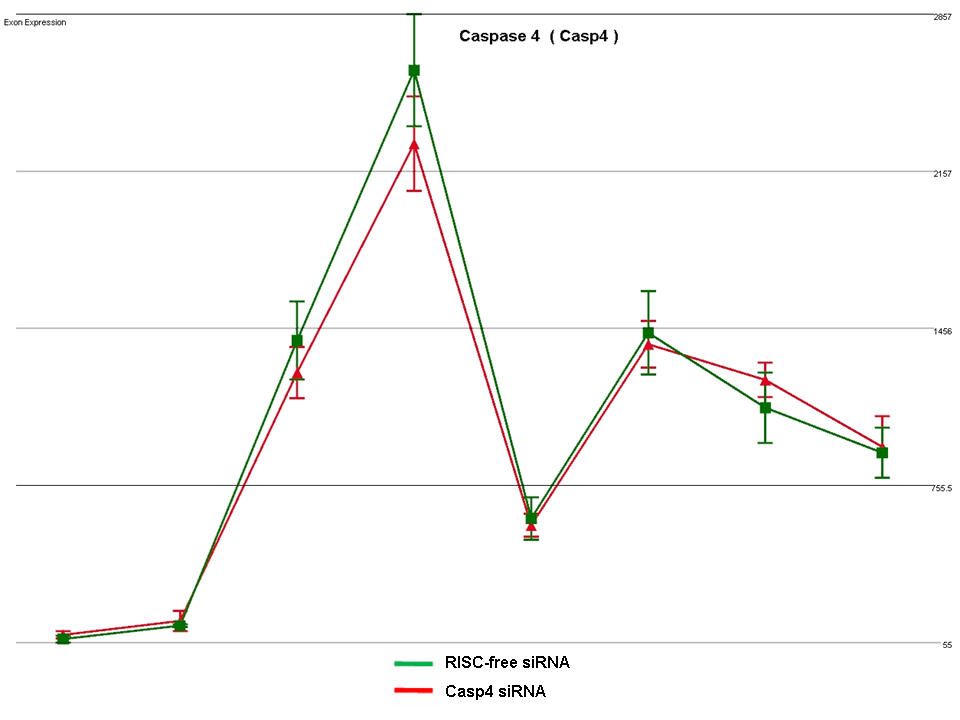

Supplement: Additional file 3 — Exon-level assessment of gene knockdown by siRNA. Level of knock-down at each of the exon probesets across the entire length of transcripts in the presence of IFNγ. Green profiles represents the median signal intensity in the three control arrays (RISC-Free) and red line the median signal intensity in the three siRNA targeted arrays. [file 1471-2164-10-372-S3.zip › Casp4.PNG]

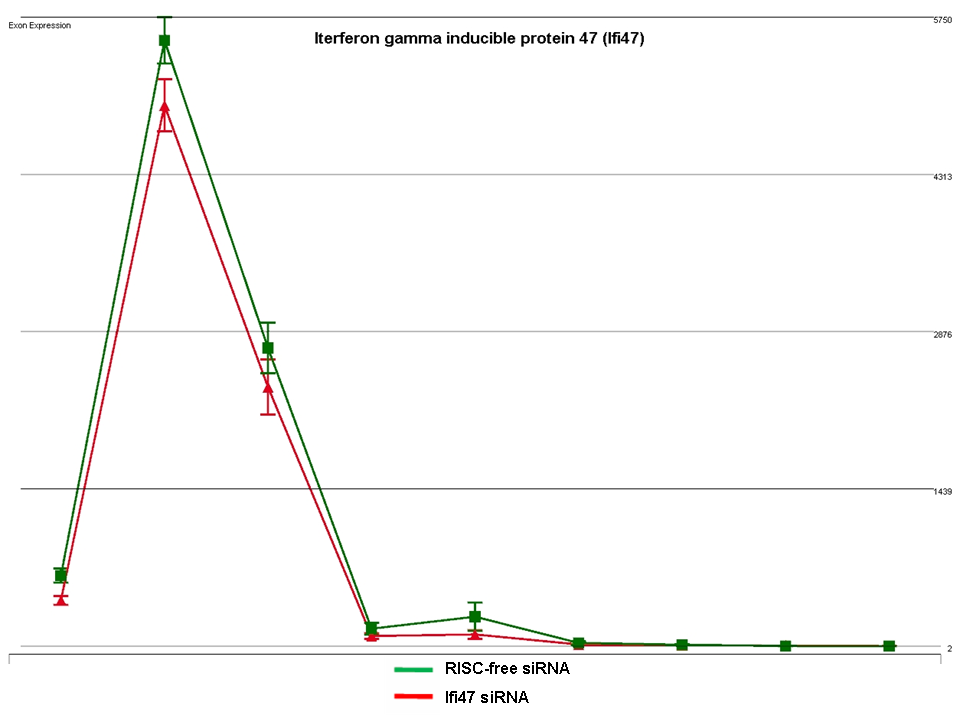

Supplement: Additional file 3 — Exon-level assessment of gene knockdown by siRNA. Level of knock-down at each of the exon probesets across the entire length of transcripts in the presence of IFNγ. Green profiles represents the median signal intensity in the three control arrays (RISC-Free) and red line the median signal intensity in the three siRNA targeted arrays. [file 1471-2164-10-372-S3.zip › Ifi47.PNG]

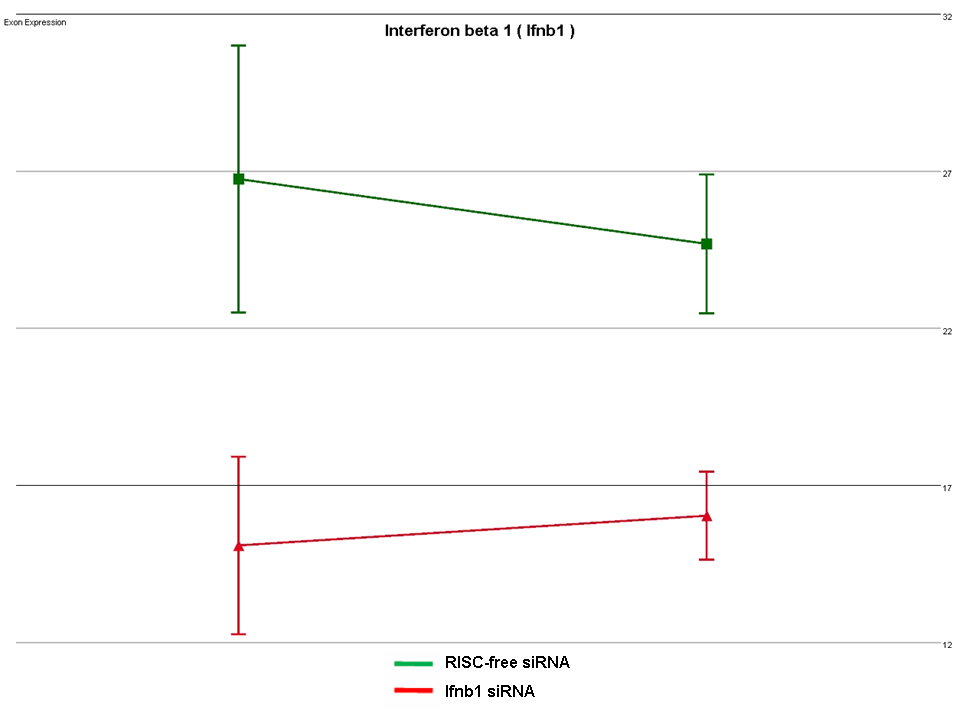

Supplement: Additional file 3 — Exon-level assessment of gene knockdown by siRNA. Level of knock-down at each of the exon probesets across the entire length of transcripts in the presence of IFNγ. Green profiles represents the median signal intensity in the three control arrays (RISC-Free) and red line the median signal intensity in the three siRNA targeted arrays. [file 1471-2164-10-372-S3.zip › Ifnb1.PNG]

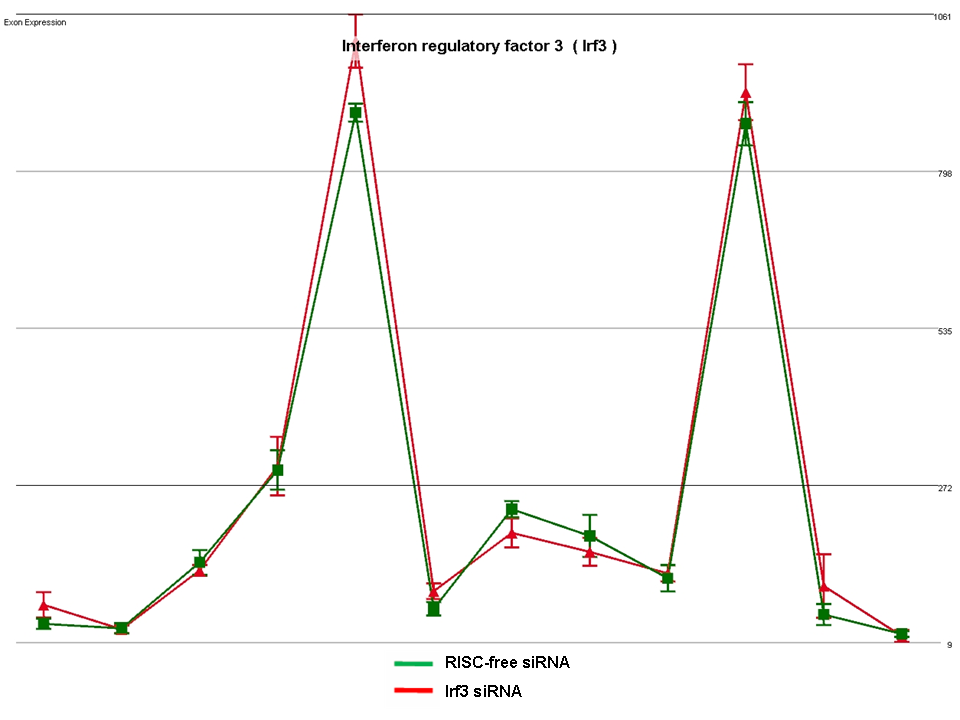

Supplement: Additional file 3 — Exon-level assessment of gene knockdown by siRNA. Level of knock-down at each of the exon probesets across the entire length of transcripts in the presence of IFNγ. Green profiles represents the median signal intensity in the three control arrays (RISC-Free) and red line the median signal intensity in the three siRNA targeted arrays. [file 1471-2164-10-372-S3.zip › Irf3.PNG]

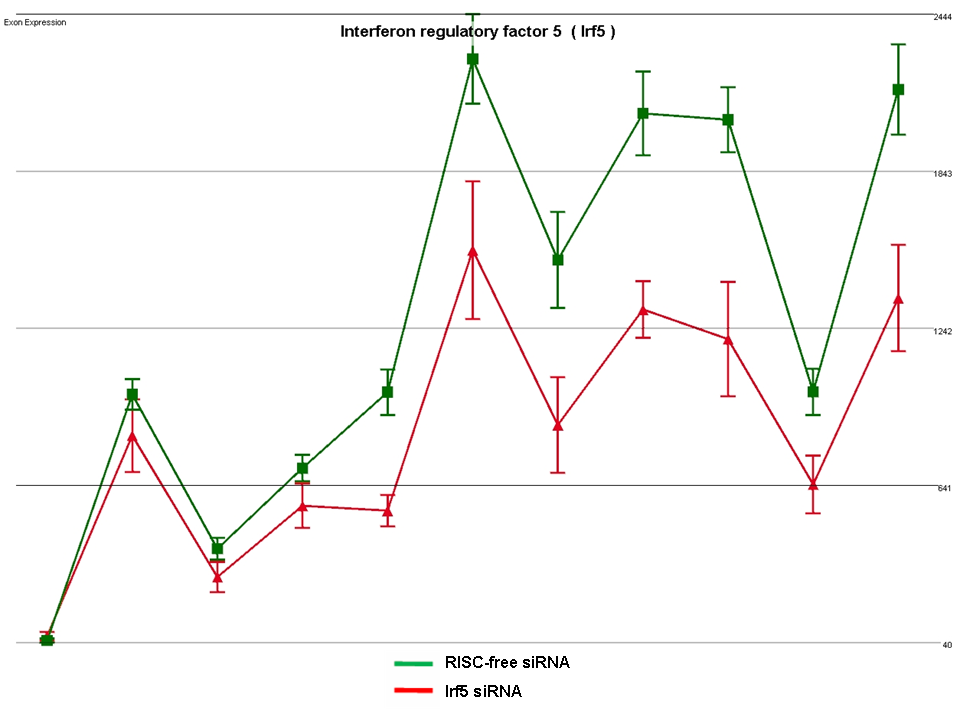

Supplement: Additional file 3 — Exon-level assessment of gene knockdown by siRNA. Level of knock-down at each of the exon probesets across the entire length of transcripts in the presence of IFNγ. Green profiles represents the median signal intensity in the three control arrays (RISC-Free) and red line the median signal intensity in the three siRNA targeted arrays. [file 1471-2164-10-372-S3.zip › Irf5.PNG]

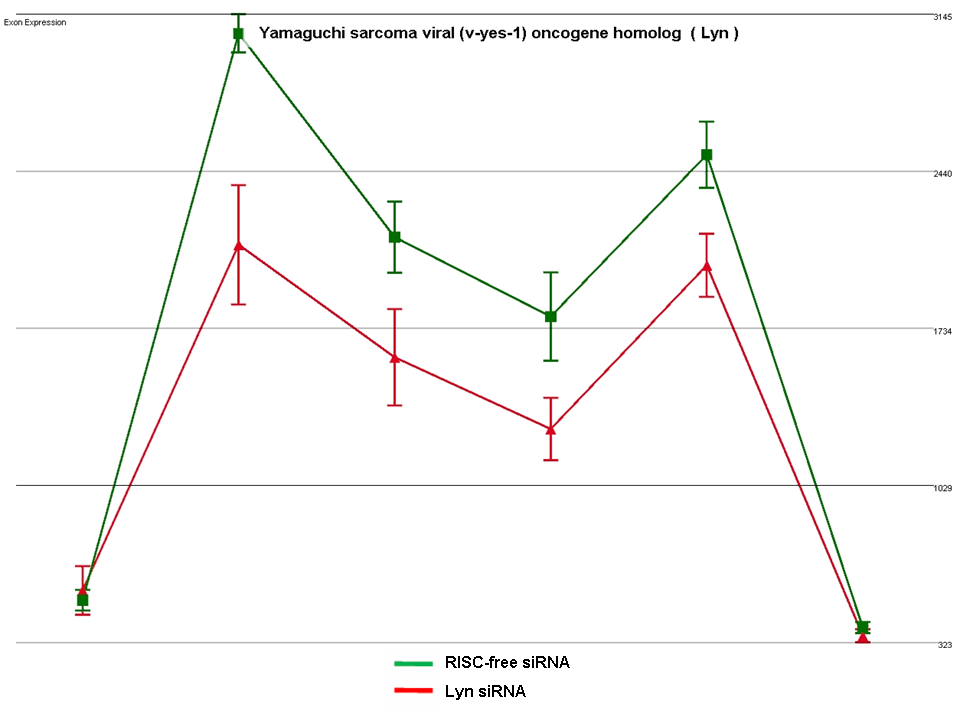

Supplement: Additional file 3 — Exon-level assessment of gene knockdown by siRNA. Level of knock-down at each of the exon probesets across the entire length of transcripts in the presence of IFNγ. Green profiles represents the median signal intensity in the three control arrays (RISC-Free) and red line the median signal intensity in the three siRNA targeted arrays. [file 1471-2164-10-372-S3.zip › Lyn.PNG]

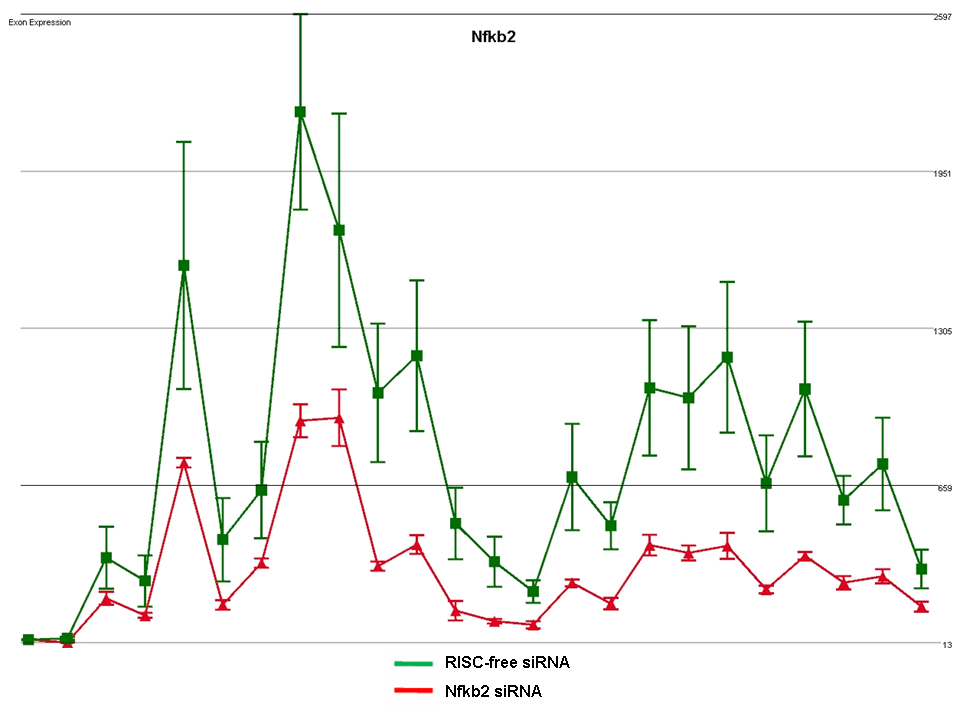

Supplement: Additional file 3 — Exon-level assessment of gene knockdown by siRNA. Level of knock-down at each of the exon probesets across the entire length of transcripts in the presence of IFNγ. Green profiles represents the median signal intensity in the three control arrays (RISC-Free) and red line the median signal intensity in the three siRNA targeted arrays. [file 1471-2164-10-372-S3.zip › Nfkb2.PNG]

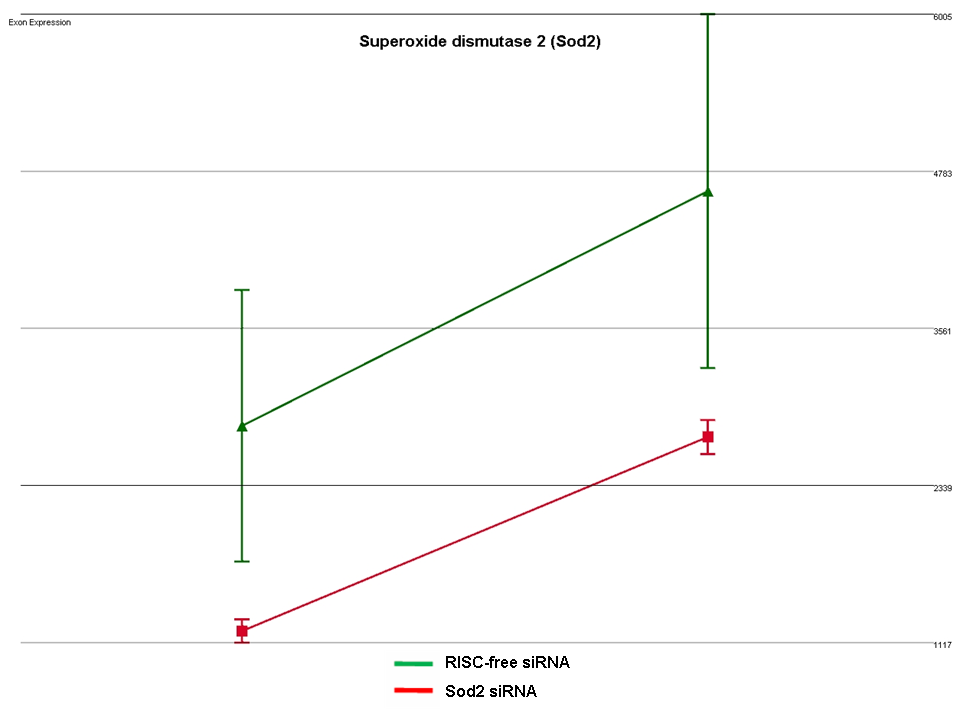

Supplement: Additional file 3 — Exon-level assessment of gene knockdown by siRNA. Level of knock-down at each of the exon probesets across the entire length of transcripts in the presence of IFNγ. Green profiles represents the median signal intensity in the three control arrays (RISC-Free) and red line the median signal intensity in the three siRNA targeted arrays. [file 1471-2164-10-372-S3.zip › Sod2.PNG]

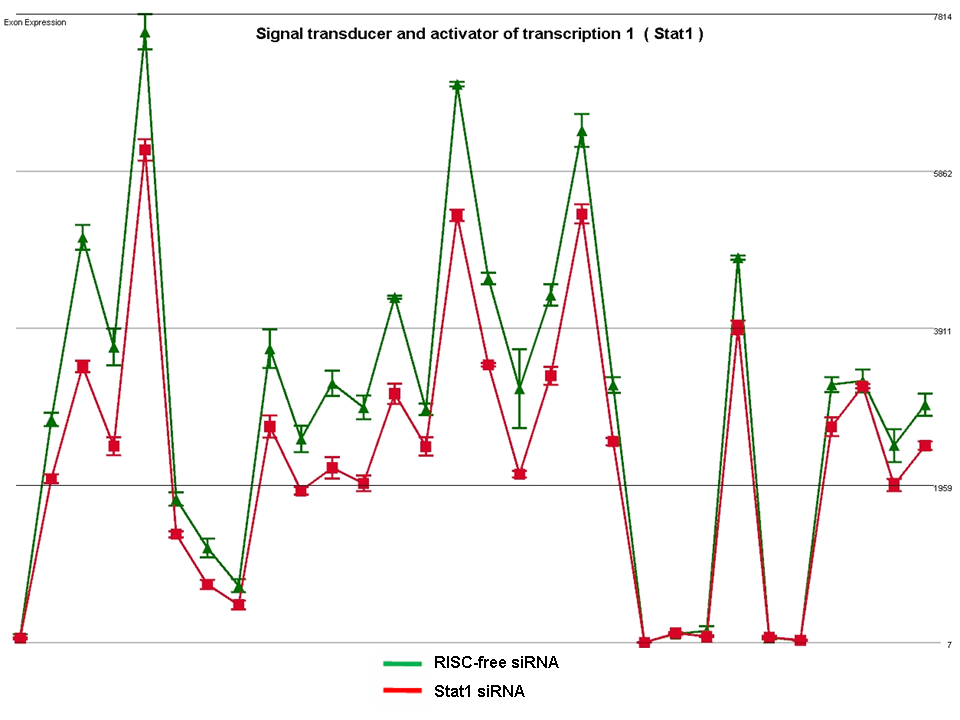

Supplement: Additional file 3 — Exon-level assessment of gene knockdown by siRNA. Level of knock-down at each of the exon probesets across the entire length of transcripts in the presence of IFNγ. Green profiles represents the median signal intensity in the three control arrays (RISC-Free) and red line the median signal intensity in the three siRNA targeted arrays. [file 1471-2164-10-372-S3.zip › Stat1.PNG]

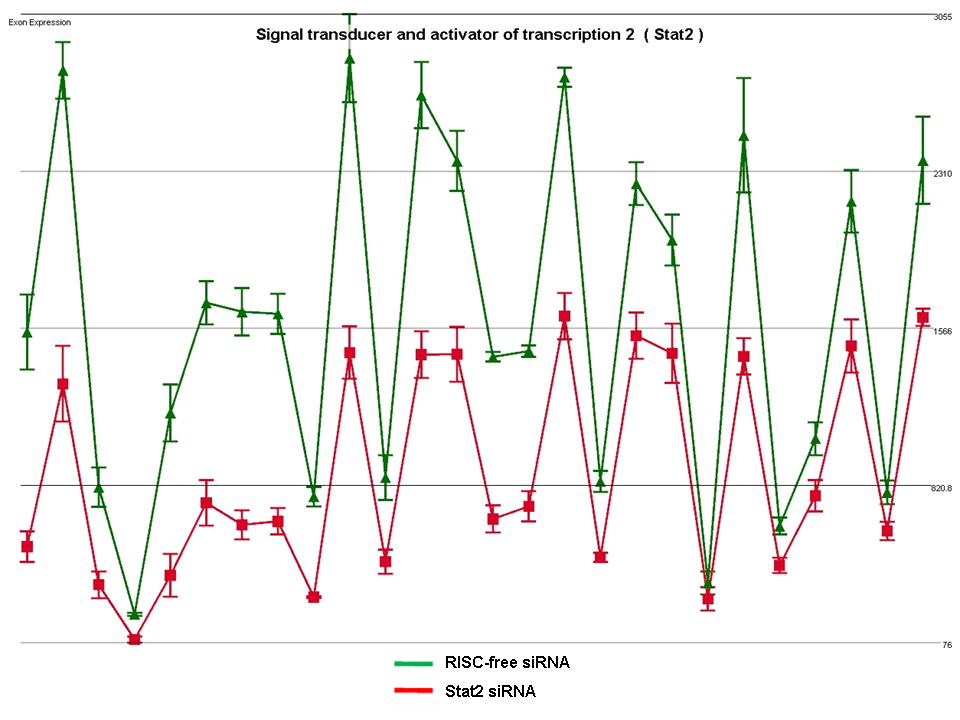

Supplement: Additional file 3 — Exon-level assessment of gene knockdown by siRNA. Level of knock-down at each of the exon probesets across the entire length of transcripts in the presence of IFNγ. Green profiles represents the median signal intensity in the three control arrays (RISC-Free) and red line the median signal intensity in the three siRNA targeted arrays. [file 1471-2164-10-372-S3.zip › Stat2.PNG]

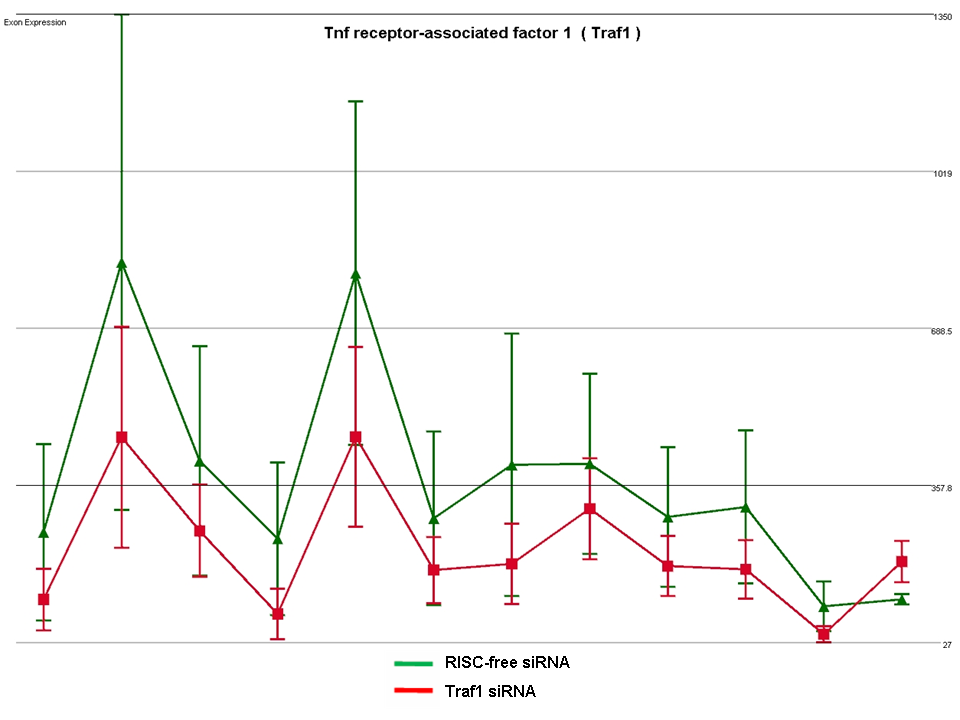

Supplement: Additional file 3 — Exon-level assessment of gene knockdown by siRNA. Level of knock-down at each of the exon probesets across the entire length of transcripts in the presence of IFNγ. Green profiles represents the median signal intensity in the three control arrays (RISC-Free) and red line the median signal intensity in the three siRNA targeted arrays. [file 1471-2164-10-372-S3.zip › Traf1.PNG]

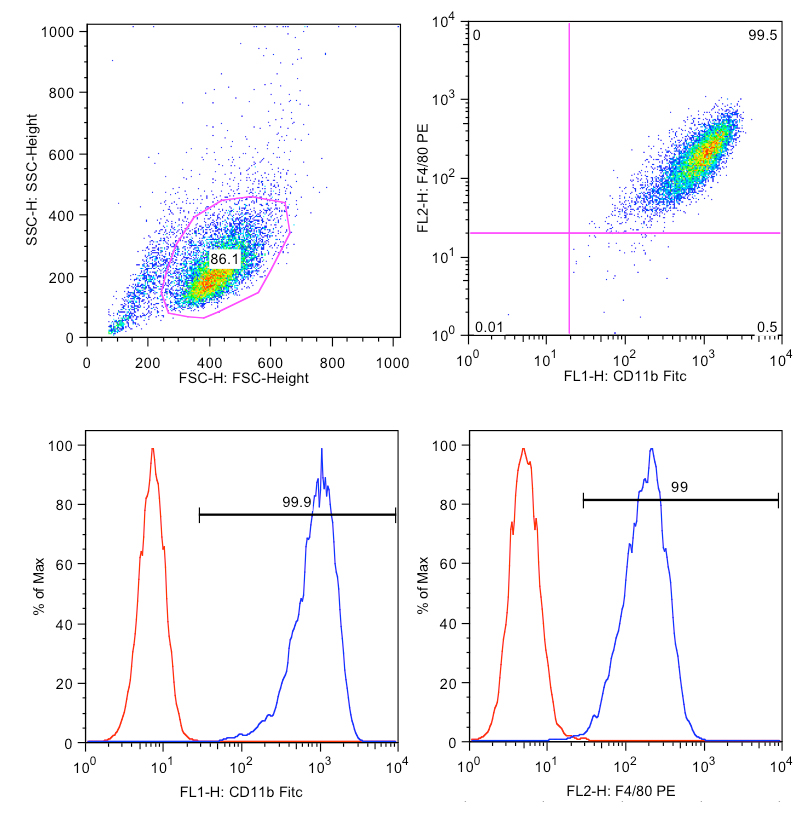

Supplement: Additional File 9 — Macrophage flow cytometry. Mouse bone-marrow derived monocytes were stained on day 6 of differentiation for CD11b and F4/80 cell surface markers to identify the presence of macrophages. Over 99% of cells within the cultures are CD11b and F4/80 positive. [file 1471-2164-10-372-S9.jpeg]
